# *In ovo* Feeding of L-Leucine Improves Antioxidative Capacity and Spleen Weight and Changes Amino Acid Concentrations in Broilers After Chronic Thermal Stress

**DOI:** 10.3389/fvets.2022.862572

**Published:** 2022-03-18

**Authors:** Guofeng Han, Yangyang Cui, Dan Shen, Mingyang Li, Yu Ren, Takashi Bungo, Vishwajit S. Chowdhury, Yansen Li, Chunmei Li

**Affiliations:** ^1^Research Center for Livestock Environmental Control and Smart Production, College of Animal Science and Technology, Nanjing Agricultural University, Nanjing, China; ^2^Department of Bioresource Science, Graduate School of Integrated Sciences for Life, Hiroshima University, Higashihiroshima, Japan; ^3^Division for Experimental Natural Science, Faculty of Arts and Science, Kyushu University, Fukuoka, Japan

**Keywords:** amino acid, *in ovo* feeding, heat stress, spleen, chicken

## Abstract

L-Leucine (L-Leu) was demonstrated to confer thermotolerance by *in ovo* feeding in broiler chicks and chickens in our previous studies. However, the L-Leu-mediated roles in recovering from the detrimental effects of heat stress in broilers are still unknown. This study aimed to investigate the effects of L-Leu *in ovo* feeding on the growth performance, relative weight of organs, serum metabolites and antioxidant parameters, and gene expression profiles in broiler chickens after chronic heat stress. Fertilized broiler eggs (Ross 308) were subjected to *in ovo* feeding of sterile water (0.5 mL/egg) or L-Leu (69 μmol/0.5 mL/egg) on embryonic day 7. After hatching, the male chicks were separated and used for the current study. All chickens were subjected to thermal stress exposure from 21 to 39 days of age and 1 week of recovery from 40 to 46 days of age. The results showed that *in ovo* feeding of L-Leu did not affect the body weight gain or relative weight of organs under chronic heat stress; however, the serum glutathione peroxidase was significantly increased and serum malondialdehyde was significantly decreased by L-Leu at 39 days of age. After 1 week of recovery, *in ovo* feeding of L-Leu significantly improved the relative spleen weight at 46 days of age. Subsequent RNA-seq analysis in the spleen showed that a total of 77 significant differentially expressed genes (DEGs) were identified, including 62 upregulated DEGs and 15 downregulated DEGs. Aspartic-type endopeptidase and peptidase activities were upregulated after recovery in the L-Leu group. The expression of genes related to B cell homeostatic proliferation and vestibular receptor cell differentiation, morphogenesis and development was downregulated in the L-Leu group. Moreover, the concentrations of serum catalase, total antioxidative capacity, isoleucine and ammonia were significantly decreased by L-Leu *in ovo* feeding after recovery. These results suggested that L-Leu *in ovo* feeding promoted the recovery of antioxidative status after chronic heat stress in broiler chickens.

## Introduction

Commercial broilers have the characteristics of fast growth and a high feed conversion rate after decades of intensive selection (1) and serve as one of the most widely consumed meats worldwide. However, chickens are very sensitive to heat stress (2, 3), as they lack sweat glands and have high metabolic rates (4). Heat stress is a critical problem that causes negative effects on the efficient and healthy production of broiler chickens (5). To mitigate the heat stress-induced negative impacts on poultry production, many studies have been conducted to propose suitable and efficient strategies to overcome this problem in broiler chickens (6, 7). The technology of *in ovo* administration was first proposed for vaccination against Marek's disease and well-developed with definition of deposition sites and injection days in 1980s (8). The technique of delivering various nutrients, supplements, immunostimulants, vaccines, and drugs via the *in ovo* route is gaining wide attention among researchers for boosting the production performance and immunity and for safeguarding the health of poultry.

In our previous studies, it was demonstrated that L-leucine (L-Leu) *in ovo* feeding afforded thermotolerance, reduced food intake and improved lipid metabolism under acute heat stress in broiler chicks (9–12). Some essential amino acids, including Leu and isoleucine (Ile), were significantly increased in the liver and decreased in the plasma by L-Leu *in ovo* administration under acute heat stress, which was considered a contributor to L-Leu-mediated thermotolerance (13). Interestingly, the daily body weight (BW) gain was also significantly higher in L-Leu *in ovo*-treated broilers than in control broilers under chronic heat stress (13). Chronic heat stress alters hypothalamic integrity and increases serum albumin, cholesterol and triglyceride (TG) levels (14, 15). After chronic heat stress, the total protein (TP), total globulin and glucose (GLU) concentrations were found to be elevated in Japanese quail (16). Partial recovery was also observed in TP, TG, blood pH, rectal temperature and respiration rate after chronic heat stress in slow-growing chicks (17). However, whether *in ovo* L-Leu-mediated thermotolerance affects the recovery process of broilers after chronic heat stress is still unclear. Thus, the first objective of the current study was to investigate the effects of L-Leu *in ovo* feeding on the growth, relative weight of organs, plasma metabolites and amino acid changes after chronic heat stress in broiler chickens.

Heat stress causes oxidative stress by producing reactive oxygen species, which damage enzyme functions and impair the immune response in broilers (5, 18). Chronic heat stress also reduces the relative weight of lymphoid organs (thymus, bursa, and spleen) in broiler chickens (19). The spleen, as the largest immune organ, plays an important role in regulating cellular and humoral immunity in poultry. Different responses were observed in the physiological and immunological parameters under chronic heat stress between different breeds of broilers, which indicated that heat tolerance was correlated with the immunological parameters under thermal stress (20). Therefore, the second aim of this study was to investigate the *in ovo* L-Leu-mediated effects on serum antioxidative parameters and the transcriptome profiles in the spleen after chronic heat stress in broiler chickens.

## Materials and Methods

### Experimental Design

A total of 320 fertilized broiler eggs (Ross 308 strain; 48-week-old parent stock) were purchased from a local hatchery in Nanjing, China. Eggs were individually weighed, numbered in pencil, and then separated into two groups (control and L-Leu; *n* = 160/group) based on egg weight to form groups as uniform as possible. The average egg weights of the control and L-Leu groups were 67.3 ± 0.3 g and 66.8 ± 0.3 g, respectively. All eggs were placed into an incubator (Hongde 2112 type incubator, Hongde Comp., Shandong, China). The incubation temperature was 37.6°C with 60–70% relative humidity and autoturning every 1.5 h. On embryonic day (ED) 7, all eggs were candled, and 23 unfertilized eggs (12 eggs from the control group and 11 eggs from the L-Leu group) were detected and discarded properly. The remaining eggs were subjected to *in ovo* injection of L-Leu solution (69 μmol/0.5 mL sterile water/egg; Beijing Solarbio Science & Technology Co., Ltd., China) or sterile water (0.5 mL/egg) for the corresponding group, as described elsewhere (9). In brief, a small hole was made at the blunt end of the egg after sterilization. L-Leu solution or sterile water was injected to a depth of 25 mm, and the small holes were immediately sealed with a glue gun (Deli Group Co., Ltd., China). After injection, the eggs were returned to the incubator. The eggs were shifted to hatching trays at the end of ED 18 as a preparation of hatching.

**Figure 1:**
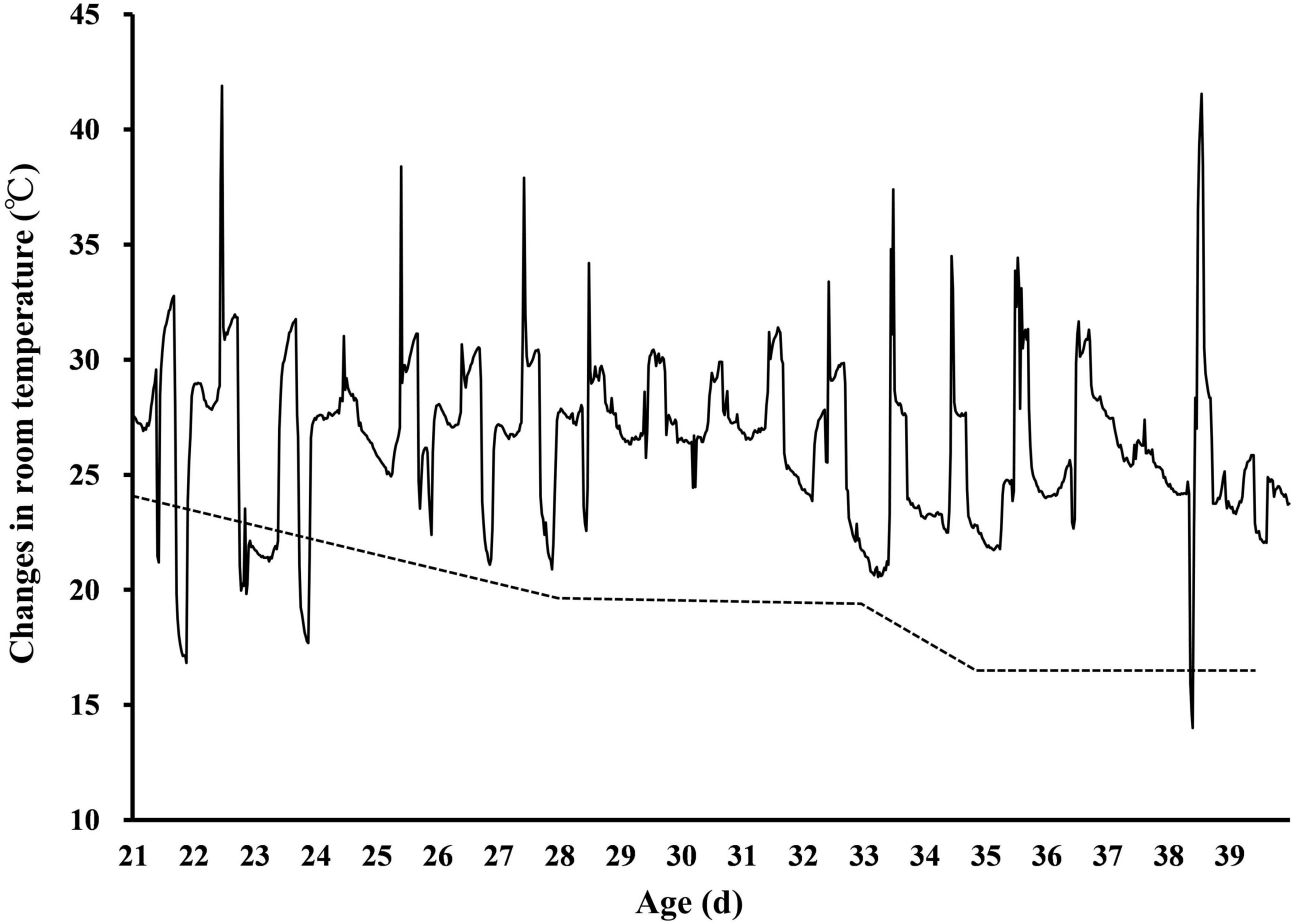
Changes in the ambient temperature of the poultry room during chronic heat stress. The dotted line indicates the standard control temperature for broiler rearing.

After hatching, chicks were housed in groups in metal cages under a controlled thermoneutral temperature (CT) following management guidelines. The room temperature was controlled with air conditions. The chicks were provided with free access to feed with commercial standard diets. All birds were under a light cycle of 23 h light plus 1 h dark (23:00–00:00) from 1 to 7 days of age and 18 h light plus 6 h dark (22:00–04:00) from 8 to 46 days of age. At 2 days of age, male chicks were selected by feather identification, as it was confirmed that *in ovo* feeding of L-Leu afforded thermotolerance in male but not female broiler chicks (11, 13). At 15 days of age, 24 chicks with similar body weights were selected from each group and equally assigned to separate cages (length × width × height: 0.9 m × 0.6 m × 0.5 m) as 8 replicates with 3 male chicks each. From 21 to 39 days of age, the birds were subjected to natural summer heat waves. The room temperature and relative humidity were recorded by a digital thermometer and hygrometer (RC-4HC type, Elitech Technology Inc., Jiangsu, China), and the room temperature results are shown in Figure 1. Food and water were provided *ad libitum* throughout the experiment. The behavior of panting was confirmed daily under heat stress. At 39 days of age, 8 birds (one bird per replicate) were randomly selected from each group for sampling after completing the heat stress exposure. The chickens were properly anesthetized with dry ice (Jiangsu Yongtai Dry Ice Co., Ltd, Taixing, China) before being killed for sample collection. Body weight was measured after anesthetization. To collect serum, blood was immediately collected from the jugular vein into ice-cold tubes and centrifuged at 10,000 × *g* at 4°C for 4 min. The heart, liver, spleen and bursa were collected and weighed by the same person without knowing the grouping information. The relative organ weight (%) of the heart, liver, spleen and bursa was expressed as the ratio of organ weight (g) to body weight (g). Spleen and liver were immediately collected following weighing and snap frozen using liquid nitrogen. All the collected samples were stored at −80°C until further analysis. After chronic heat stress, the birds were kept under CT conditions for recovery from 40 to 46 days of age. At 46 days of age, 8 birds (one bird per replicate) were randomly selected from each group, and the measurement, sample processing and storage were the same as those at 39 days of age.

This study was performed according to the Guidelines for the Care and Use of Laboratory Animals prepared by the Institutional Animal Care and Use Committee of Nanjing Agricultural University [permit number SYXK (Su) 2011-0036].

### RNA Isolation, Library Construction, and Sequencing

To study the transcriptome profiles in the spleen after 1 week recovery from chronic thermal stress, spleen samples of 46-day old broilers (*n* = 3/group) were applied transcriptome analysis. Total RNA was extracted using TRIzol (Invitrogen, CA, USA) following the manufacturer's instructions. The RNA purity and integrity were checked by a NanoPhotometer^®^ spectrophotometer (IMPLEN, CA, USA) and an RNA Nano 6000 Assay Kit of the Bioanalyzer 2100 system (Agilent Technologies, CA, USA). Then, the concentration of RNA was determined with a Qubit^®^ RNA Assay Kit in a Qubit^®^2.0 Flurometer (Life Technologies, CA, USA). A total amount of 3 μg RNA per sample was used as input material for the RNA sample preparations. Sequencing libraries were generated using the NEBNext^®^ UltraTM RNA Library Prep Kit for Illumina^®^ (NEB, USA) following the manufacturer's recommendations, and index codes were added to attribute sequences to each sample. PCR products were purified (AMPure XP system, Beckman Coulter, CA, USA), and library quality was assessed on the Agilent Bioanalyzer 2100 system. Clustering of the index-coded samples was performed on a cBot Cluster Generation System using TruSeq PE Cluster Kit v3-cBot-HS (Illumina) according to the manufacturer's instructions. After cluster generation, the library preparations were sequenced on an Illumina platform, and 125/150 bp paired-end reads were generated.

### Quality Control, Transcriptome Assembly and Bioinformatics Analyses

Raw data (raw reads) in fastq format were first processed through in-house Perl scripts. In this step, clean data (clean reads) were obtained by removing reads containing adapters, reads containing poly-N and low-quality reads from the raw data. At the same time, the Q20, Q30, and GC contents of the clean data were calculated. All downstream analyses were based on clean data with high quality. Reference genome and gene model annotation files were downloaded from the genome website directly. The mapped reads of each sample were assembled by StringTie (v1.3.3b) in a reference-based approach. FeatureCounts v1.5.0-p3 was used to count the read numbers mapped to each gene.

Differential expression analysis was performed using the DESeq2 R package (1.16.1), based on the same RNA samples of spleen of 46-day old broilers. Gene Ontology (GO) enrichment analysis of differentially expressed genes was implemented by the clusterProfiler R package, in which gene length bias was corrected. GO terms with corrected *P* < 0.05 were considered significantly enriched by differentially expressed genes. KEGG is a database resource for understanding the high-level functions and utilities of biological systems, such as cells, organisms and ecosystems, from molecular-level information, especially large-scale molecular datasets generated by genome sequencing and other high-throughput experimental technologies (http://www.genome.jp/kegg/). We used the clusterProfiler R package to test the statistical enrichment of differentially expressed genes in KEGG pathways.

### Analysis of Serum Free Amino Acids, Metabolites and Antioxidative Parameters

The serum free amino acid concentrations were analyzed using a fully automatic amino acid analyzer (L-8080 type, Hitachi, Japan) according to the method described elsewhere (21). The serum was well mixed with a 5% sulfonic acid solution for deproteinization. After 30 min, the serum samples were centrifuged at 4°C and 20,000 × *g* for 20 min. The supernatant was collected and filtered using a 0.22-μm filter (Biosharp, Guangzhou Saiguo Biotech Co., Ltd., Guangzhou, China). The filtrate and standard solution were incorporated into the amino acid analyzer. The amino acid concentrations were expressed as pmol/μL in the serum. Since the system used here could not separate the L- and D-forms of the amino acids, only the names of the amino acids are used in the results of the determined amino acids.

Serum concentrations of GLU, TP, uric acid (UA), total cholesterol (T-CHO), non-esterified fatty acid (NEFA) and TG were measured by a biochemical automatic analyzer (Hitachi 7020, Hitachi, Tokyo, Japan) as described elsewhere (22).

The serum concentrations of malondialdehyde (MDA), superoxide dismutase (SOD), glutathione peroxidase (GPx), catalase (CAT) activity and total antioxidant capacity (T-AOC) were analyzed by the corresponding assay kits (Nanjing Jiancheng Bioengineering Institute, Nanjing, China) according to the instructions of the manufacturer as described elsewhere (23).

### Statistical Analysis

The body weight, organ indices, serum metabolites, antioxidative parameters and amino acid levels were statistically analyzed using a Student's *t-*test. A *P* < 0.05 was used to denote significant differences. Statistical analysis was conducted using GraphPad Prism 6 (GraphPad Software, Inc., San Diego, CA, USA). The results are expressed as the mean ± standard error of the mean (SEM). The number of chickens used for statistical analysis in each group is shown in the figure legends and table notes.

## Results

### Changes in Body Weight and Organ Indices After Chronic Heat Stress

After chronic heat stress, the body weight and organ indices were not affected by L-Leu treatment at 39 days of age. After 1 week of recovery, L-Leu *in ovo* feeding did not improve the body weight; similarly, the weight and relative weights of the liver, heart and bursa were also not affected by L-Leu treatment. Interestingly, the relative spleen weight was significantly (*P* < 0.05) increased by L-Leu *in ovo* feeding after chronic heat stress at 46 days of age (Table 1).

**Table 1:** The changes in body weight and (relative) organ weight after chronic heat stress (39-day old) and 1 week recovery (46-day old) in broiler chickens.

	**BW**	**Liver**	**Heart**	**Spleen**	**Bursa**
**39-day old**
Control	1,693 ± 95	43.23 ± 1.71	10.03 ± 0.74	2.23 ± 0.29	4.18 ± 0.30
L-Leu	1,699 ± 55	44.84 ± 1.01	9.51 ± 0.39	2.61 ± 0.02	4.09 ± 0.39
*P*-value	NS	NS	NS	NS	NS
**46-day old**
Control	2,646 ± 167	57.27 ± 3.55	12.63 ± 0.75	3.32 ± 0.20	6.14 ± 0.47
L-Leu	2,388 ± 135	53.78 ± 2.29	10.71 ± 0.60	4.55 ± 0.42	5.59 ± 0.63
*P*-value	NS	NS	NS	NS	NS
**Relative weight (%)**
**39-day old**
Control		2.61 ± 0.19	0.59 ± 0.04	0.14 ± 0.02	0.25 ± 0.02
L-Leu		2.65 ± 0.08	0.56 ± 0.01	0.16 ± 0.02	0.24 ± 0.03
*P*-value		NS	NS	NS	NS
**46-day old**
Control		2.18 ± 0.09	0.48 ± 0.02	0.13 ± 0.01 ^b^	0.24 ± 0.02
L-Leu		2.27 ± 0.09	0.45 ± 0.01	0.20 ± 0.03 ^a^	0.23 ± 0.03
*P*-value		NS	NS	P <0.05	NS

*The number of replicates used in each group was as n = 7–8. The unit for body weight and organ weight is g. Different superscripts in the same row indicate significant differences (P < 0.05) between treatments. Values are means ± SEM; L-Leu, L-leucine; BW, body weight; NS, not significant*.

### Transcriptomic Analysis Overview

Six cDNA libraries were constructed, and 131.76 and 143.48 million raw sequence reads were generated from the control and L-Leu libraries, respectively. After removing the low-quality reads, 122.58 and 132.21 million clean reads were retained with 94.64–95.27% Q30 bases and 46.28–47.49% GC content, respectively. The correlation of gene expression levels between samples is an important indicator to test the reliability of the experiment and the accuracy of the sample selection. The correlation analysis shows that the *R*^2^ between the biological duplicates in this study was higher than 0.95, as shown in Supplementary Figure 1.

**Figure 2:**
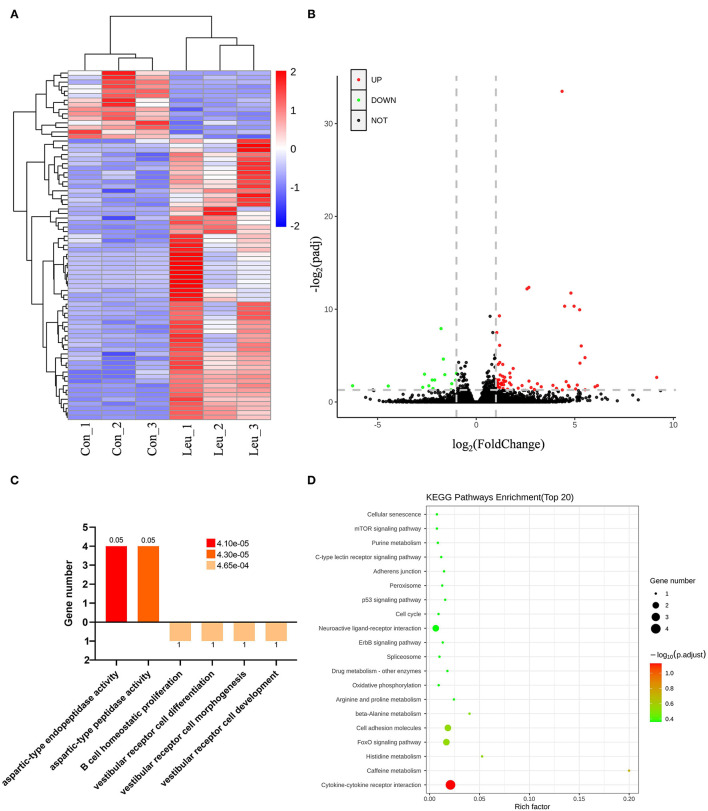
Transcriptomic analysis of the spleens of the control (Con) and L-leucine (L-Leu) groups. **(A)** Heatmap showing the significant differentially expressed genes (DEGs); **(B)** Volcano plots showing the DEGs between the Con and L-Leu groups; **(C,D)** Functional classification of identified DEGs by GO enrichment analysis **(C)** and KEGG enrichment analysis **(D)** between the Con and L-Leu groups.

A total of 21,527 unigenes were detected in the current study. Among these unigenes, 77 were identified as differentially expressed genes (DEGs) with padj <0.05 and Ilog2foldchangeI > 1, including 62 upregulated and 15 downregulated genes in the L-Leu group vs. the control group. This information is presented in a hierarchical clustering and a volcano plot (Figures 2A,B).

### Functional Classification of Identified DEGs

As a significant difference was found in the relative spleen weight between the control and L-Leu groups after recovery from heat stress, enrichment GO terms were analyzed for the DEGs to evaluate the effects of L-Leu *in ovo* treatment on spleen recovery. After 1 week of recovery, the upregulated DEGs were associated with molecular function (MF), aspartic-type endopeptidase activity and aspartic-type peptidase activity. In contrast, downregulated DEGs were associated with biological process (BP), including B cell homeostatic proliferation, vestibular receptor cell differentiation, vestibular receptor cell morphogenesis and vestibular receptor cell development (Figure 2C). Subsequently, KEGG analysis was applied to reflect the transcriptional changes in metabolic pathways. A total of 23 pathways were enriched, including cytokine–cytokine receptor interaction and caffeine metabolism (*P* < 0.05). However, no significantly enriched pathway [false discovery rate (FDR) <0.05] was identified between the two groups (Figure 2D).

### Changes in Serum Antioxidative Parameters, Free Amino Acids and Metabolites After Chronic Heat Stress

**Figure 3:**
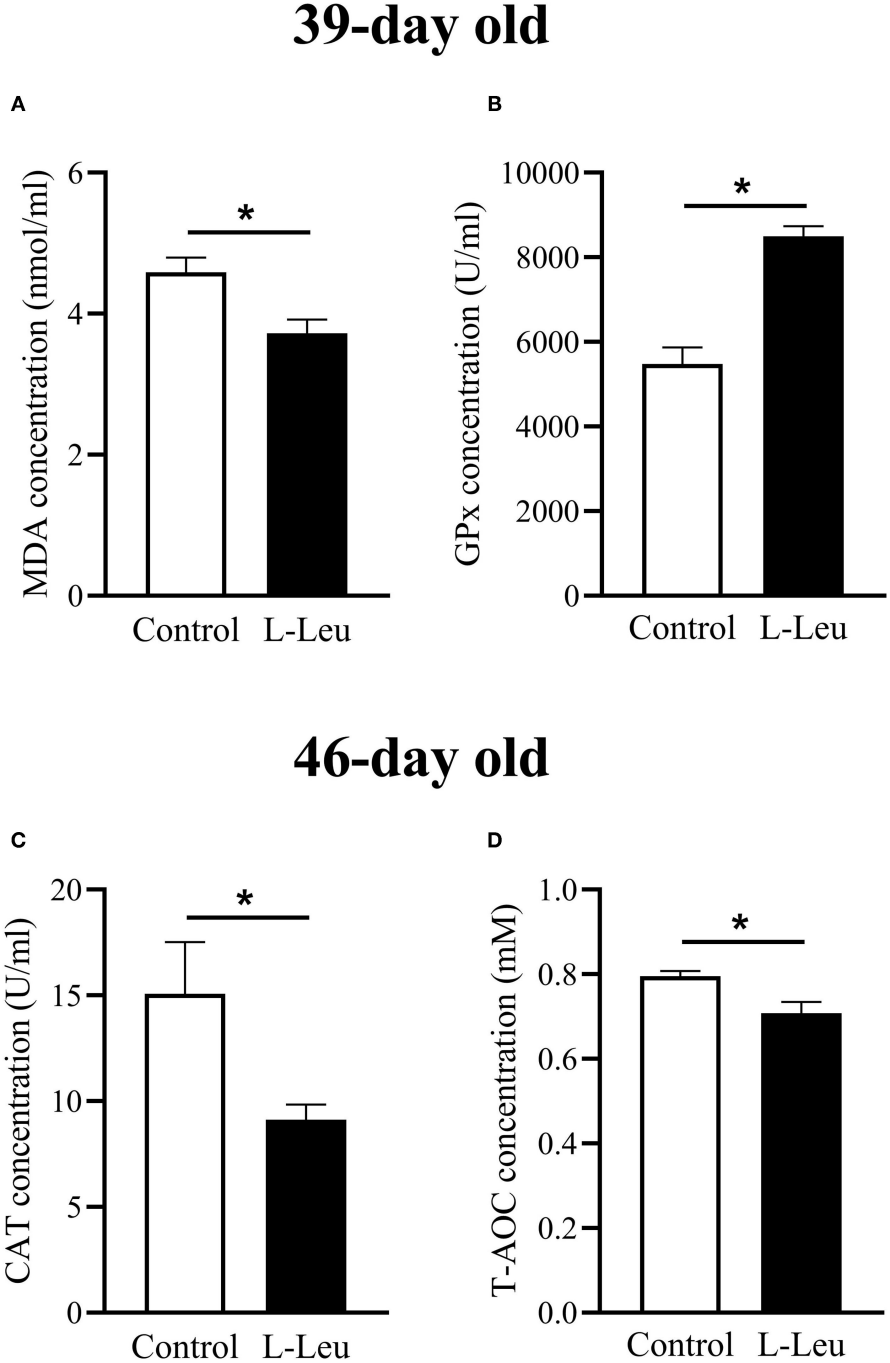
Changes in serum antioxidative parameters in the control and L-leucine (L-Leu) groups. **(A,B)** Serum concentrations of malondialdehyde (MDA) and glutathione peroxidase (GPx) after chronic heat stress at 39 days of age; **(C,D)** serum concentrations of catalase (CAT) activity and total antioxidant capacity (T-AOC) after 1 week of recovery at 46 days of age. The number of chickens in each group was *n* = 6. *P < 0.05.

**Figure 4:**
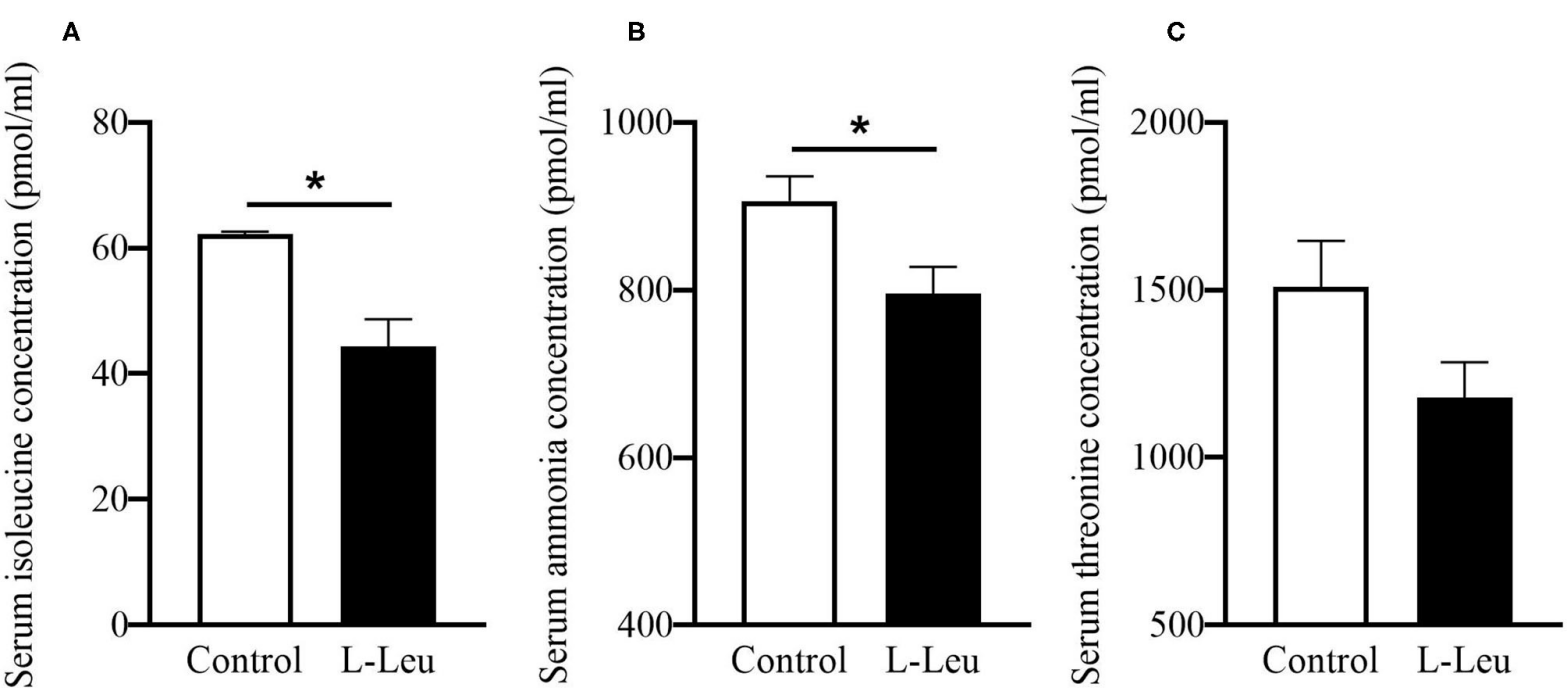
Effects of L-leucine (L-Leu) *in ovo* feeding on serum isoleucine **(A)**, ammonia **(B)**, and threonine **(C)** in male broiler chickens after 1 week of recovery at 46 days of age. The number of chickens in each group was *n* = 6. *P < 0.05.

The serum concentrations of TP, GLU, UA, T-CHO, TG, and NEFA were not affected by L-Leu *in ovo* treatment after chronic heat stress or 1 week of recovery (Supplementary Table 1). However, L-Leu *in ovo* feeding caused a significant decrease in serum MDA and an increase in serum GPx at 39 days of age (Figures 3A,B). After 1 week of recovery, serum CAT and T-AOC concentrations were significantly decreased by L-Leu treatment at 46 days of age (Figures 3C,D). Moreover, L-Leu *in ovo* feeding caused a significant reduction in serum Ile and ammonia concentrations and a decreasing trend in serum threonine (*P* = 0.087) at 46 days of age (Figure 4).

## Discussion

Chronic heat stress, cyclic or continuous, reduces food intake, increases food conversion ratios, and retards growth in broiler chickens (6). Recently, we reported that L-Leu *in ovo* feeding improved growth performance under chronic heat stress, especially during its cyclic period (13). However, body weight was not affected by L-Leu *in ovo* feeding after chronic heat stress in the current study. Our previous studies demonstrated that *in ovo* injection of L-Leu afforded thermotolerance in broiler chicks and chickens, as the body temperature increase was suppressed by L-Leu under acute heat stress (9, 11, 13). However, the difference in body temperature between control and L-Leu-treated birds narrowed after 3 h of thermal exposure and disappeared after 6 h under continued high ambient temperature in neonatal broiler chicks (*unpublished data*). The L-Leu-mediated improvement in body weight gain was also diminished under continuous chronic heat stress in broiler chickens from 30 to 44 days of age (13). A dietary supply of some antioxidant minerals was reported to decrease the food conversion ratio without improving growth under chronic heat stress (24). However, the final body mass can be significantly improved when supplied with a high dose of antioxidant minerals under chronic heat stress (25). Therefore, the current results indicated that L-Leu-mediated thermotolerance was limited for promoting growth under chronic heat stress, especially a continuous pattern of heat stress.

It was demonstrated that heat stress causes impairment of the immune system, and chronic heat stress is considered to increase heat-related maladies in poultry (26). The spleen is an important peripheral lymphoid tissue (27), and the relative weight of the spleen is reduced under chronic heat stress (28). In the current study, the relative spleen weight was not affected by L-Leu treatment after chronic heat exposure. It was expected that chronic heat stress would cause a reduction in the relative spleen weight in both groups. However, the relative spleen weight in the L-Leu group was significantly higher than that in the control group after 1 week of recovery in the current study. Supplementation with additives, including zinc, ascorbic acid and chromium, was reported to increase spleen weight and improve the immune status of broilers reared under heat stress (18, 29). The significantly increased relative spleen weight indicated that the heat stress-related depression in immunocompetence was reduced by L-Leu *in ovo* treatment in the current study. Combined with the results of body weight changes, it could be suggested that L-Leu-mediated thermotolerance was not enough to support immunity during chronic heat stress, at least for supporting peripheral lymphoid tissues, in broiler chickens. However, the recovery of immune function could be expected to be promoted by L-Leu *in ovo* feeding after ending the heat exposure. Future studies will clarify this matter with an investigation of immune parameters in serum and spleen tissue under chronic heat stress.

To determine the possible mechanism for spleen recovery after chronic heat stress, splenic transcriptome profiles were conducted in this study. Overall, the aspartic-type endopeptidase and peptidase activities were enhanced in the L-Leu *in ovo* group. Endopeptidase and peptidase are known to break peptide bonds between amino acids and catalyze the hydrolysis of a peptide bond. Previous studies found that amino acid metabolism was modified by L-Leu *in ovo* treatment under acute or chronic heat stress (10, 13). B cell homeostatic proliferation and vestibular receptor cell development in the spleen were downregulated by L-Leu treatment, which might be feedback to the improved splenic cell proliferation during recovery. In birds, B cells mature in the bursa and emigrate to the spleen after hatching (30). However, the bursa weight was not affected in the current study. Moreover, serum immunoglobulin A and G were also not affected by L-Leu treatment after recovery (*data not shown*). In the current study, quantitative real-time PCR was not conducted to confirm the DEGs, and future studies will clarify the mechanisms of changing splenic weight after chronic heat stress.

Thermal stress causes an increase in body temperature and accelerates metabolism (31). The energy requirement for maintain homeostasis is increased, which stimulates glucose metabolism and suppresses lipid metabolism under heat stress (2, 32). Previous study supported that L-Leu *in ovo* feeding causes to activate lipid metabolism and affords thermotolerance in broiler chicks under acute heat stress (11). However, the serum metabolites, includes glucose, TG, UA, etc., were not affected by L-Leu *in ovo* feeding after chronic heat stress in this study. It is agreed with our previous results under chronic heat stress (13), as the strategies for coping with heat stress might also be different between short- and long-term patterns in broilers (33). Heat stress is a significant cause of economic losses in poultry production through a decrease in growth, and impacts on the physiological status of poultry by inducing oxidative stress in the body. Heat stress has been confirmed to induce oxidative stress *in vivo* or *in vitro* (34, 35). Reactive oxygen species (ROS) are byproducts of oxygen metabolism and are continuously produced in all aerobic organisms. Enhanced ROS disturbs mitochondrial homeostasis and induces lipid peroxidation. GPx is a ubiquitous intracellular enzyme that breaks down lipid hydroperoxide by utilizing lipid peroxide as a substrate. It has been reported that a decrease in GPx activity is associated with an imbalance between oxidative stress and antioxidants (36). The serum GPx concentration was significantly increased by L-Leu *in ovo* feeding under chronic heat stress, which suggested that heat stress-induced lipid peroxidation might be attenuated in L-Leu-treated broilers. MDA is the principal product of polyunsaturated fatty acid peroxidation, and heat stress results in higher mitochondrial and plasma levels of MDA (37). L-Leu *in ovo* administration has been shown to reduce serum MDA levels after chronic heat stress, which indicated that heat stress-induced oxidative damage is reduced in L-Leu-treated birds, as plasma MDA is considered a biomarker of oxidative stress (38).

Heat stress is associated with modified CAT activity, which is one of the key antioxidant enzymes. An *in vitro* study demonstrated that heat stress caused oxidative damage with a decreased CAT activity, and betaine treatment attenuated the heat stress-mediated oxidative damage with enhanced CAT activity (35). Moreover, serum T-AOC is considered an important parameter for assessing oxidative status (39), as T-AOC considers the cumulative effect of all antioxidants in blood and body fluids. In the current study, the decreased CAT and T-AOC concentrations in L-Leu-treated birds indicated that the oxidative status was, at least, ameliorated in comparison with the control group after 1 week of recovery. Thus, it could suggest that L-Leu *in ovo* feeding promoted broiler chickens to recover after chronic heat exposure. Similarly, acute heat stress (35°C for 3 h) induced a significant increase in ROS and antioxidative enzymes (SOD, CAT, GPx), and the above parameters gradually approached preheat levels after 12 h of recovery (40, 41). Chronic heat stress causes heavy damage and takes a long time for recovery. It was reported that 3 days of recovery was not enough for quails to fully recover from a prior 9 days of heat stress exposure (16). However, it was not clear whether the L-Leu-treated broiler chickens fully recovered after 1 week of recovery. Future studies will clarify this matter with prolonged measurements compared with one non-stresses group.

Amino acids serve as building blocks of protein. Recent studies have demonstrated that some amino acids also play important roles in the regulation of body temperature and food intake. Previous studies investigated the amino acid profiles following L-Leu *in ovo* injection in embryos and heat-exposed chicks or chickens (10, 13, 21). This is the first study to clarify the amino acid changes in L-Leu-injected chickens after recovery from heat stress. L-Leu *in ovo* injection was shown to cause a significant increase in hepatic Ile and a decrease in plasma Ile after chronic heat stress (13). Dietary Ile supplementation was reported to improve the immune response and alleviate rotavirus infection in piglets (42). After 1 week of recovery, the decreased serum Ile in the L-Leu group indicated that the immune response was defused, as L-Leu-treated birds were expected to recover quickly compared with the control chickens. The lower serum ammonia in the L-Leu-treated group suggested that ammonia might be utilized to synthesize certain biomolecules (43). In previous reports, L-Leu *in ovo* administration was shown to decrease plasma ammonia during embryogenesis, which may cause a prenatal imprinting on ammonia-related metabolism (11). Interestingly, the serum Leu concentration was not affected by *in ovo* feeding of L-Leu after 1 week of recovery. Similar results showed that the blood Leu concentrations in embryos and heat-exposed chicks were also not affected by L-Leu feeding *in ovo* (10, 21). These results suggested that L-Leu is a trigger rather than a long-term regulator of L-Leu-mediated thermotolerance, as our trial experiments showed that central or oral administration of L-Leu had no effects on body temperature regulation in chicks.

## Conclusions

In summary, L-Leu *in ovo* feeding reduced oxidative damage and improved antioxidative ability and relative spleen weight after chronic heat stress, which suggested that L-Leu promoted the recovery process in heat-exposed broiler chickens. The amino acid Ile was expected to be one of the contributors to the L-Leu-mediated benefit during recovery. Future studies will confirm the effects of dietary Ile on heat-exposed broilers.

## Data Availability Statement

The original contributions presented in the study are included in the article/Supplementary Material, further inquiries can be directed to the corresponding author/s.

## Ethics Statement

The animal study was reviewed and approved by Institutional Animal Care and Use Committee of Nanjing Agricultural University.

## Author Contributions

GH and CL designed this research. GH, YC, DS, and ML conducted the animal experiment. GH, YC, YR, and YL performed the sample analysis and statistical analysis. GH and VC wrote the manuscript. TB and CL reviewed and edited the manuscript. All authors read and approved the final manuscript.

## Funding

This work was partly supported by grants from the Jiangsu Agricultural Industry Technology System (grant no. JATS [2021] 480) and the National Nature Science Foundation of China (grant no. 32072781) to CL.

## Conflict of Interest

The authors declare that the research was conducted in the absence of any commercial or financial relationships that could be construed as a potential conflict of interest.

## Publisher's Note

All claims expressed in this article are solely those of the authors and do not necessarily represent those of their affiliated organizations, or those of the publisher, the editors and the reviewers. Any product that may be evaluated in this article, or claim that may be made by its manufacturer, is not guaranteed or endorsed by the publisher.

## Supplementary Material

Supplementary Figure 1 The results of the correlation analysis among the samples.

Supplementary Table 1 The changes in serum metabolites after chronic heat stress (39-day old) and 1 week recovery (46-day old) in broiler chickens.
