# Supplementary figures and images for: In ovo Feeding of L-Leucine Improves Antioxidative Capacity and Spleen Weight and Changes Amino Acid Concentrations in Broilers After Chronic Thermal Stress
Source: Front Vet Sci. 2022 Mar 18;9:862572. doi: 10.3389/fvets.2022.862572 (PMC8971722; doi:10.3389/fvets.2022.862572)

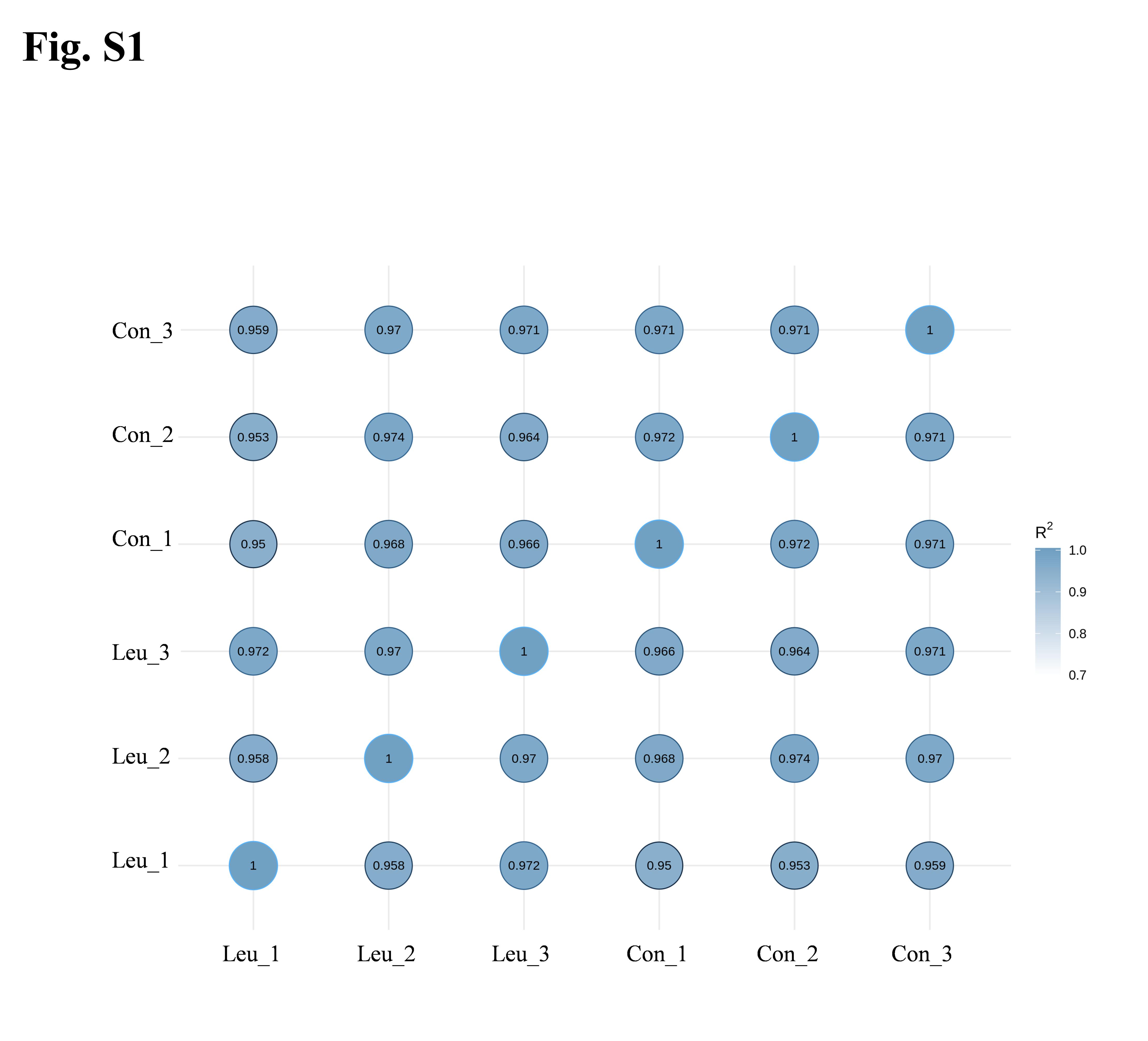

Supplement: Supplementary Figure 1 — The results of the correlation analysis among the samples. [file Image_1.JPEG]
