# Supplementary material for: In ovo Feeding of L-Leucine Improves Antioxidative Capacity and Spleen Weight and Changes Amino Acid Concentrations in Broilers After Chronic Thermal Stress
Source: Front Vet Sci. 2022 Mar 18;9:862572. doi: 10.3389/fvets.2022.862572 (PMC8971722; doi:10.3389/fvets.2022.862572)
Supplement: Supplementary Table 1 — The changes in serum metabolites after chronic heat stress (39-day old) and 1 week recovery (46-day old) in broiler chickens. [file Table_1.docx]

Table S1. The changes in serum metabolites after chronic heat stress (39-day old) and one-week recovery (46-day old) in broiler chickens

|  | TP | GLU | UA | T-CHO | TG | NEFA |
| --- | --- | --- | --- | --- | --- | --- |
| 39-day old |  |  |  |  |  |  |
| Control | 33.4 ± 0.9 | 12.6 ± 0.6 | 309 ± 48 | 3.4 ± 0.2 | 0.67 ± 0.09 | 0.68 ± 0.05 |
| L-Leu | 34.2 ± 1.1 | 13.0 ± 0.7 | 355 ± 47 | 3.1 ± 0.2 | 0.72 ± 0.10 | 0.56 ± 0.03 |
| *P*-value | NS | NS | NS | NS | NS | NS |
| 46-day old |  |  |  |  |  |  |
| Control | 32.7 ± 1.1 | 11.9 ± 0.4 | 408 ± 22 | 3.1 ± 0.1 | 1.33 ± 0.29 | 0.51 ± 0.02 |
| L-Leu | 35.3 ± 1.9 | 11.5 ± 0.5 | 438 ± 56 | 2.9 ± 0.2 | 0.83 ± 0.12 | 0.50 ± 0.03 |
| *P*-value | NS | NS | NS | NS | NS | NS |

The number in each group was as n = 7-8. Values are means ± SEM; L-Leu, L-leucine; TP, total protein (g/L); GLU, glucose (mmol/L); UA, uric acid (µmol/L); T-CHO, total cholesterol (mmol/L); TG, triglyceride (mmol/L); NEFA, non-esterified fatty acid (mmol/L); NS, not significant.
